# Declines in Prevalence of Human Papillomavirus Vaccine-Type Infection Among Females after Introduction of Vaccine — United States, 2003–2018

**DOI:** 10.15585/mmwr.mm7012a2

**Published:** 2021-03-26

**Authors:** Hannah G. Rosenblum, Rayleen M. Lewis, Julia W. Gargano, Troy D. Querec, Elizabeth R. Unger, Lauri E. Markowitz

**Affiliations:** ^1^Epidemic Intelligence Service, CDC, ^2^Division of Viral Diseases, National Center for Immunization and Respiratory Diseases, CDC, ^3^Synergy America, Inc., Duluth, Georgia; ^4^Division of High-Consequence Pathogens and Pathology, National Center for Emerging and Zoonotic Infectious Diseases, CDC.

Human papillomavirus (HPV) is the most common sexually transmitted infection in the United States (*1*). Although most infections resolve without clinical sequalae, persistent HPV infection can cause cervical, other anogenital, and oropharyngeal cancers and anogenital warts. HPV vaccination has been recommended in the United States at age 11–12 years since 2006 for females and since 2011 for males. Catch-up vaccination is recommended through age 26 years.* A quadrivalent vaccine (4vHPV) targeting types 6, 11, 16, and 18 was mainly used until 2015, when a 9-valent vaccine (9vHPV), targeting the same four types as 4vHPV and five additional types (31, 33, 45, 52, and 58), was introduced; 9vHPV has been the only vaccine available in the United States since the end of 2016 (*2*). HPV vaccination coverage has increased but remains lower than that of other vaccinations recommended for adolescents (*3*). A decrease in prevalence of 4vHPV types detected in cervicovaginal swabs among young females from the prevaccine era (2003–2006) to 2007–2010 in the National Health and Nutrition Examination Survey (NHANES) was an early indicator of vaccine impact (*2*) and was also observed in later periods (*4*,*5*). NHANES data from 2017–2018 were included in this analysis to update HPV prevalence estimates among females aged 14–34 years. From the prevaccine era to 2015–2018, significant decreases in 4vHPV-type prevalence occurred among females aged 14–19 years (88%) and 20–24 years (81%). In sexually experienced females, 4vHPV-type prevalence decreased in those who reported receiving ≥1 HPV vaccine dose (97% among those aged 14–19 years, 86% among those aged 20–24 years) and in those who reported no vaccination (87% among those aged 14–19 years, 65% among those aged 20–24 years). Significant declines among unvaccinated females suggest herd effects. These data show increasing impact of HPV vaccination in the United States. HPV vaccination is a critical prevention tool against HPV infection, anogenital warts, and HPV-attributable precancers and cancers. HPV vaccination is highly effective and is recommended routinely at age 11–12 years and through 26 years for persons not already vaccinated.

NHANES is an ongoing cross-sectional survey conducted by CDC’s National Center for Health Statistics designed to monitor the health and nutrition of the U.S. non-institutionalized civilian population. Data collection for NHANES was approved by the National Center for Health Statistics Research Ethics Review Board. This activity was reviewed by CDC and was conducted consistent with applicable federal law and CDC policy.^†^Demographic and HPV vaccination information are obtained during in-home interviews. Sexual behavior information is obtained via audio computer-assisted self-interview, and self-collected cervicovaginal specimens are obtained in mobile examination centers.^§^ CDC determines HPV DNA genotypes from these specimens using L1 consensus polymerase chain reaction testing followed by type-specific hybridization to detect 37 HPV types^¶^ and β-globin (*6*). Samples negative for both HPV and β-globin are considered inadequate and were excluded from analysis. Data from 2003 through 2018 were analyzed in 4-year periods: 2003–2006, 2007–2010, 2011–2014, and 2015–2018. Age groups were categorized as 14–19, 20–24, 25–29, and 30–34 years. Self-reported race/ethnicity was categorized as non-Hispanic White, non-Hispanic Black, Mexican American,** or other races. Self- or parent-reported vaccination history was analyzed as ever vaccinated (receipt of ≥1 dose). Sexual behaviors analyzed included ever having had sex^††^ (sexually experienced) and number of lifetime sex partners (fewer than three or three or more partners^§§^). HPV prevalences^¶¶^ and 95% confidence intervals (CIs) were estimated for each 4-year period for the following type categories: 4vHPV types,*** five additional 9vHPV types,^†††^ non-4vHPV types,^§§§^ and non-9vHPV types.^¶¶¶^ Prevalence ratios (PRs) and adjusted prevalence ratios (aPRs), comparing 2015–2018 with 2003–2006, were estimated using logistic regression. For females aged 14–19 years, estimates were adjusted a priori for race/ethnicity and ever having had sex. For females aged 20–24, 25–29, and 30–34 years, estimates were adjusted a priori for race/ethnicity and number of lifetime sex partners. Distributions of participant characteristics and behaviors were estimated for each 4-year period; 2015–2018 was compared with 2003–2006 using Wald F tests. Among sexually experienced females, 4vHPV-type prevalence in each 4-year period was estimated overall, by age group and vaccination history. Complex survey analytic methods were used to account for the survey design; analyses used examination weights.**** Statistical significance was defined as p<0.05, or 95% CIs that did not include one for PRs. Prevalence estimates with relative standard error >30% were noted for stability concerns. Data management and analysis were performed using SAS (version 9.4; SAS Institute) and SUDAAN (version 11.0; RTI International). Because the analyses required restricted-use data, the data were accessed through CDC’s Research Data Center.^††††^

Prevalence of 4vHPV-type infection decreased 88% among females aged 14–19 years, from 11.5% during 2003–2006 (prevaccine era) to 1.1% during 2015–2018 (aPR = 0.12; 95% CI = 0.06–0.26) (Table 1). Prevalence of five additional 9vHPV types in this age group decreased 65%, from 8.4% during 2003–2006 to 2.3% during 2015–2018 (aPR = 0.35; 95% CI = 0.18–0.65). Among females aged 20–24 years, 4vHPV-type prevalence decreased 81%, from 18.5% during 2003–2006 to 3.3% during 2015–2018 (aPR = 0.19; 95% CI = 0.09–0.40); the prevalence of the five additional 9vHPV types did not decline significantly. Among females in older age groups, no statistically significant differences in the prevalence of 4vHPV or the five additional 9vHPV types were noted from 2003–2006 to 2015–2018. Regarding HPV types not targeted by vaccine, among females aged 14–19 years, the prevalence of non-4vHPV types decreased from 31.2% during the prevaccine era to 20.9% during 2015–2018 (aPR = 0.72; 95% CI = 0.57–0.92); the prevalence of non-9vHPV types decreased from 29.0% to 20.6% (aPR = 0.77; 95% CI = 0.61–0.98). The prevalences of non-4vHPV and non-9vHPV types in the other age groups were not statistically significantly different during 2015–2018 compared with 2003–2006.

**TABLE 1:** Prevalence of human papillomavirus (HPV) infection among females aged 14–34 years, by age group and survey years — National Health and Nutrition Examination Survey, United States, 2003–2018*

Age group (yrs) and HPV types	Prevaccine era 2003–2006	2007–2010	2011–2014	2015–2018	Comparison of 2015–2018 with 2003–2006	
	% (95% CI)				PR (95% CI)	aPR (95% CI)^†^
**14–19**	n = 1,363	n = 740	n = 797	n = 666	0.10 (0.05–0.21)	0.12 (0.06–0.26)
4vHPV^§^	11.5 (9.1–14.4)	5.0 (3.8–6.6)	3.3 (1.9–5.8)	1.1 (0.5–2.4) ^¶^		
Additional five types in 9vHPV**	8.4 (6.6–10.6)	6.1 (4.4–8.5)	5.3 (3.4–8.4)	2.3 (1.3–4.1)	0.28 (0.15–0.51)	0.35 (0.18–0.65)
Non-4vHPV^††^	31.2 (27.9–34.8)	25.3 (21.4–29.5)	25.5 (21.3–30.2)	20.9 (16.9–25.6)	0.67 (0.53–0.84)	0.72 (0.57–0.92)
Non-9vHPV^§§^	29.0 (26.0–32.2)	24.4 (20.8–28.4)	24.7 (20.6–29.4)	20.6 (16.6–25.3)	0.71 (0.57–0.90)	0.77 (0.61–0.98)
**20–24**	n = 432	n = 445	n = 442	n = 368	0.18 (0.09–0.35)	0.19 (0.09–0.40)
4vHPV^§^	18.5 (14.9–22.8)	19.9 (15.4–25.3)	7.2 (4.7–11.1)	3.3 (1.7–6.3) ^¶^		
Additional five types in 9vHPV**	16.5 (11.3–23.4)	13.8 (10.2–18.2)	13.2 (8.8–19.4)	10.2 (7.2–14.4)	0.62 (0.38–1.02)	0.62 (0.38–1.01)
Non-4vHPV^††^	50.7 (43.4–58.0)	57.4 (51.3–63.3)	55.8 (49.9–61.6)	49.9 (42.3–57.5)	0.98 (0.80–1.21)	0.97 (0.80–1.18)
Non-9vHPV^§§^	47.6 (40.7–54.6)	54.9 (48.9–60.8)	53.4 (47.8–58.8)	47.1 (39.7–54.7)	0.99 (0.80–1.22)	0.97 (0.79–1.18)
**25–29**	n = 403	n = 414	n = 395	n = 430	0.77 (0.46–1.29)	0.85 (0.50–1.46)
4vHPV^§^	11.8 (8.8–15.6)	13.1 (10.0–17.2)	8.8 (6.3–12.1)	9.1 (5.8–14.0)		
Additional five types in 9vHPV**	10.8 (7.3–15.7)	13.1 (9.7–17.3)	13.9 (10.5–18.1)	11.6 (8.1–16.3)	1.07 (0.64–1.79)	0.99 (0.58–1.67)
Non-4vHPV^††^	43.8 (38.9–48.9)	48.6 (43.7–53.6)	43.7 (37.7–49.9)	45.2 (39.2–51.4)	1.03 (0.87–1.23)	1.05 (0.86–1.27)
Non-9vHPV^§§^	39.8 (34.8–45.0)	44.7 (40.0–49.4)	42.0 (36.2–48.0)	42.1 (36.6–47.9)	1.06 (0.88–1.27)	1.07 (0.88–1.31)
**30–34**	n = 389	n = 433	n = 433	n = 413	0.65 (0.38–1.11)	0.67 (0.37–1.21)
4vHPV^§^	9.5 (6.7–13.2)	8.9 (6.5–11.9)	7.1 (5.1–9.9)	6.2 (4.0–9.5)		
Additional five types in 9vHPV**	9.8 (7.1–13.5)	6.8 (4.7–9.9)	6.9 (4.6–10.0)	6.9 (4.4–10.8)	0.70 (0.41–1.21)	0.68 (0.37–1.27)
Non-4vHPV^††^	44.5 (39.1–50.1)	37.8 (31.6–44.5)	39.2 (33.6–45.0)	34.7 (29.1–40.8)	0.78 (0.63–0.96)	0.82 (0.67–1.00)
Non-9vHPV^§§^	40.4 (35.0–46.0)	36.1 (30.3–42.3)	38.2 (32.7–44.0)	31.9 (26.6–37.6)	0.79 (0.64–0.98)	0.83 (0.67–1.03)

**Abbreviations**: aPR = adjusted prevalence ratio; CI = confidence interval; 4vHPV = quadrivalent HPV vaccine; 9vHPV = 9-valent HPV vaccine; PR = prevalence ratio.

* All analyses were weighted using the National Health and Nutrition Examination Survey examination sample weights.

^†^ Adjustments for aPR: females aged 14–19 years, race/ethnicity and ever had sex; females aged 20–24, 25–29, and 30–34 years, race/ethnicity and number of lifetime sex partners (fewer than three or three or more).

^§^ HPV 6, 11, 16, or 18.

^¶^ Relative standard error >30% and ≤50%, considered unstable.

** HPV 31, 33, 45, 52, or 58.

^††^ Thirty-three HPV types detected using linear array that are not HPV 6, 11, 16, or 18.

^§§^ Twenty-eight HPV types detected using linear array that are not HPV 6, 11, 16, 18, 31, 33, 45, 52, or 58.

Reported receipt of ≥1 HPV vaccine dose increased from 2007–2010 to 2015–2018 in all age groups (Table 2). The percentages of females aged 14–19 years reporting ever having had sex and three or more lifetime partners were significantly lower during 2015–2018 than during 2003–2006. Among females in older age groups, during 2003–2006 and 2015–2018, >90% reported ever having had sex; the percentage with three or more lifetime partners did not change significantly. The distribution of race/ethnicity was different in most age groups during 2015–2018 than during the prevaccine era.

**TABLE 2:** Characteristics of females aged 14–34 years, by age group and survey years — National Health and Nutrition Examination Survey, United States, 2003–2018*

Age group (yrs) and characteristic	% (95% CI)			
	Prevaccine era 2003–2006	2007–2010	2011–2014	2015–2018
**14–19**					
HPV vaccine history: ≥1 dose^†^	N/A	34.1 (28.4–40.3)	54.7 (49.6–59.7)	54.3 (49.2–59.4)
Ever had sex^§^	54.0 (50.9–57.0)	50.3 (45.0–55.6)	48.2 (43.0–53.3)	45.4 (41.0–49.8)
Three or more lifetime partners^§^	25.6 (22.5–29.0)	22.6 (19.9–25.5)	23.4 (19.4–27.9)	17.5 (14.3–21.2)
Race/Ethnicity^§^					
White, non-Hispanic	65.5 (58.9–71.6)	60.1 (54.4–65.6)	57.7 (50.2–64.9)	51.6 (43.3–59.8)
Black, non-Hispanic	14.8 (11.0–19.7)	15.3 (12.1–19.0)	14.5 (10.2–20.3)	14.6 (10.7–19.4)
Mexican American	10.1 (7.6–13.5)	12.4 (8.8–17.2)	13.6 (10.6–17.5)	15.1 (11.1–20.2)
Other races	9.5 (6.8–13.1)	12.2 (9.3–15.9)	14.1 (11.3–17.5)	18.7 (15.0–23.1)
**20–24**					
HPV vaccine history: ≥1 dose^†^	N/A	17.8 (12.4–24.9)	43.0 (36.0–50.4)	59.9 (53.0–66.5)
Ever had sex	91.4 (86.1–94.8)	91.9 (88.3–94.5)	91.4 (86.9–94.5)	94.9 (90.4–97.3)
Three or more lifetime partners	60.7 (53.7–67.2)	71.8 (66.1–77.0)	68.4 (63.9–72.5)	67.5 (62.3–72.4)
Race/Ethnicity					
White, non-Hispanic	61.6 (54.6–68.2)	56.7 (49.0–64.2)	55.7 (46.9–64.1)	55.3 (47.5–62.7)
Black, non-Hispanic	15.7 (11.1–21.9)	15.9 (12.3–20.5)	16.6 (11.4–23.6)	13.1 (9.1–18.6)
Mexican American	11.5 (8.2–15.9)	12.9 (9.0–18.0)	9.6 (6.6–13.8)	13.0 (8.2–20.1)
Other races	11.2 (7.7–15.9)	14.5 (10.3–19.9)	18.1 (13.8–23.4)	18.6 (14.2–23.9)
**25–29**					
HPV vaccine history: ≥1 dose^†^	N/A	7.8 (5.5–11.1)	24.8 (19.7–30.7)	40.7 (34.6–47.1)
Ever had sex	95.0 (91.7–97.1)	95.6 (92.0–97.7)	95.4 (92.5–97.2)	94.1 (89.8–96.7)
Three or more lifetime partners	73.2 (66.4–79.0)	71.9 (66.7–76.6)	71.8 (64.9–77.8)	72.1 (65.7–77.6)
Race/Ethnicity^§^					
White, non-Hispanic	65.0 (57.9–71.6)	63.6 (56.0–70.5)	56.4 (47.8–64.7)	53.8 (48.0–59.5)
Black, non-Hispanic	12.5 (8.9–17.4)	12.3 (8.7–17.2)	11.9 (8.6–16.2)	15.7 (11.5–21.1)
Mexican American	12.1 (8.6–16.7)	10.0 (7.2–13.6)	11.5 (7.6–16.9)	10.9 (6.9–16.7)
Other races	10.4 (7.1–14.9)	14.2 (10.4–19.0)	20.2 (15.9–25.4)	19.7 (15.3–25.0)
**30–34**					
HPV vaccine history: ≥1 dose^†^	N/A	3.7 (2.0–6.7)	7.0 (4.6–10.4)	18.9 (13.9–25.2)
Ever had sex	98.4 (95.4–99.4)	97.4 (93.0–99.1)	99.1 (97.8–99.7)	95.5 (91.7–97.7)
Three or more lifetime partners	73.6 (68.2–78.4)	69.3 (63.5–74.6)	75.9 (70.0–81.0)	72.3 (66.2–77.6)
Race/Ethnicity^§^					
White, non-Hispanic	61.8 (55.6–67.7)	58.1 (48.5–67.0)	59.7 (53.1–66.0)	53.4 (44.8–61.8)
Black, non-Hispanic	15.8 (11.6–21.2)	14.4 (9.9–20.5)	13.6 (10.2–18.0)	12.7 (9.2–17.1)
Mexican American	11.9 (9.4–15.1)	14.0 (9.8–19.7)	11.4 (7.4–17.4)	9.7 (6.5–14.4)
Other races	10.4 (6.8–15.7)	13.5 (9.3–19.2)	15.2 (12.6–18.2)	24.3 (18.5–31.2)

**Abbreviations:** CI = confidence interval; N/A = not applicable.

* All analyses were weighted using the National Health and Nutrition Examination Survey examination sample weights.

^†^ Significant difference from 2015–2018 to 2007–2010 (p<0.05).

^§^ Significant difference from 2015–2018 to 2003–2006 (p<0.05).

Among sexually experienced females, the prevalence of 4vHPV-type infections among those aged 14–19 years decreased from 19.3% during 2003–2006 to the following during 2015–2018: overall, 1.5% (PR = 0.08; 95% CI = 0.03–0.22); those vaccinated, 0.6% (PR = 0.03; 95% CI = 0.00–0.25); those unvaccinated, 2.4% (PR = 0.13; 95% CI = 0.03–0.48) (i.e., a 97% decrease among those vaccinated and an 87% decrease among those unvaccinated). In sexually experienced females aged 20–24 years, 4vHPV-type prevalence decreased from 17.9% during 2003–2006 to 2.5% (PR = 0.14; 95% CI = 0.05–0.41) among those vaccinated and to 6.3% (PR = 0.35; 95% CI = 0.14–0.85) among those unvaccinated during 2015–2018 (i.e. an 86% decrease among those vaccinated and a 65% decrease among those unvaccinated). Smaller, nonsignificant decreases in 4vHPV-type prevalences were observed among vaccinated females in older age groups: from 12.4% to 4.6% among those aged 25–29 years and from 9.0% to 4.4% among those aged 30–34 years (Figure) (Supplementary Table, https://stacks.cdc.gov/view/cdc/104147).

**FIGURE:**
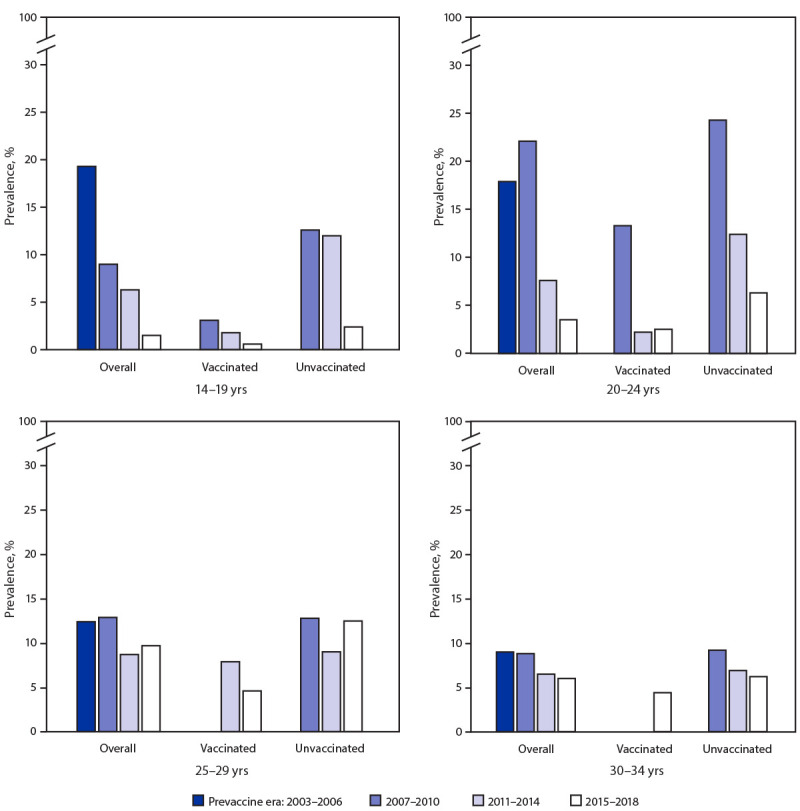
Quadrivalent vaccine-type (4vHPV-type) prevalence among sexually experienced females aged 14–34 years, by age group, vaccination history,* and survey years — National Health and Nutrition Examination Survey, United States, 2003–2018^†^^,^^§^ * Reported receipt of ≥1 HPV vaccine dose. ^†^ All analyses were weighted using the examination sample weights. ^§^ Estimates are not shown for vaccinated females aged 25–29 years in 2007–2010 and for vaccinated females aged 30–34 years in 2007–2010 and 2011–2014 because of the low proportion of females in those age groups who were vaccinated.

## Discussion

During 2015–2018, the prevalence of 4vHPV types was 88% lower than that during the prevaccine era among females aged 14–19 years and 81% lower among those aged 20–24 years after adjustment for sexual behavior and race/ethnicity. The decline among females aged 14–19 years was first observed within 4 years of vaccine introduction (*2*), and prevalence has continued to decline in subsequent years (*4*). The decline among females aged 20–24 years was first observed within 6 years of vaccine introduction (*6*); 10 years after introduction, through 2016, 4vHPV-type prevalence among women aged 20–24 years had decreased 71% (*5*). This report, through 12 years after vaccine introduction, shows sustained low 4vHPV-type prevalence among females aged 14–19 years and larger declines among those aged 20–24 years.

Very few participants surveyed during 2017–2018 would have received 9vHPV; therefore, it is likely too early for 9vHPV vaccination to explain all of the decline in prevalence of the additional 9vHPV-type infections among females aged 14–19 years. The significant declines also in prevalences of non–4vHPV-type and non–9vHPV-type infections in this age group from the prevaccine era to 2015–2018 suggest lower exposure to HPV; this is consistent with the decrease in reported sexual behaviors from 2003–2006 to 2015–2018 in the current report. Lower exposure might have contributed to some of the observed decrease in 4vHPV-type prevalence among females aged 14–19 years. Among females aged 20–24 years, an age group with no significant changes in reported sexual behavior, a dramatic decline in 4vHPV-type prevalence from the prevaccine era to 2015–2018 occurred without significant change in non–4vHPV-type or non–9vHPV-type prevalence, demonstrating vaccine impact in the absence of potential changes in HPV exposure.

In addition to significantly lower 4vHPV-type prevalence among sexually experienced vaccinated females compared with those in the prevaccine era, 4vHPV-type prevalence was also lower among unvaccinated females: 87% in females aged 14–19 years and 65% in those aged 20–24 years. These findings suggest strong herd effects, or indirect protection of unvaccinated females, as reported in previous NHANES analyses and in data from other countries (*4*,*7*). The herd effects are likely attributable to less circulation of vaccine-type HPV because of both female and male vaccination in the United States (*2*,*3*).

The findings in this report are subject to at least three limitations. First, differences in sexual behavior were noted among females aged 14–19 years in 2015–2018 compared with 2003–2006. To account for this, prevalence ratios were adjusted for sexual behaviors, and a subanalysis was restricted to sexually experienced females; however, residual confounding might be present. Second, self- and parent-reported vaccination history could have resulted in misclassification, which might bias findings, including those related to herd effects (*8*). Finally, small sample size resulted in limited precision for certain subgroup analyses.

This report adds to the robust data on the impact of the national HPV vaccination program, including herd effects. In addition to decreases in the prevalence of vaccine types, decreasing rates of cervical precancers and anogenital warts also have been demonstrated in the United States and other countries after introduction of HPV vaccination (*7*,*9*). The COVID-19 pandemic has the potential to reverse gains made in HPV vaccination coverage in the United States, as indicated by lower adolescent vaccine orders in 2020 (*3*,*10*). Efforts are needed to increase HPV vaccination to maintain the substantial progress of the vaccination program. Continued monitoring in NHANES will provide information to evaluate changes in U.S. vaccination recommendations and 9vHPV vaccine introduction on HPV prevalence as well as any setbacks attributable to the COVID-19 pandemic. HPV vaccination is a critical prevention tool against HPV infection, anogenital warts, and HPV-attributable precancers and cancers. HPV vaccination is highly effective and is recommended routinely at age 11–12 years and through age 26 years for persons not already vaccinated.

**Summary**

**What is already known about this topic?**

Through 2016, human papillomavirus (HPV) vaccine-type prevalence decreased among young females after introduction of HPV vaccination in 2006.

**What is added by this report?**

Nationally representative data through 2018 indicate that HPV vaccine-type prevalence continues to decline among females aged 14–19 (88%) and 20–24 (81%) years compared with before vaccination. The findings also show evidence of indirect protection of unvaccinated females through herd effects in these age groups.

**What are the implications for public health practice?**

HPV vaccination is a critical prevention tool against HPV infection, anogenital warts, and HPV-attributable precancers and cancers. HPV vaccination is highly effective and is recommended routinely at age 11–12 years and through age 26 years for persons not already vaccinated.

## Footnotes

*
https://www.cdc.gov/vaccines/hcp/acip-recs/vacc-specific/hpv.html#recs

† 45 C.F.R. part 46.102(l)(2), 21 C.F.R. part 56; 42 U.S.C. Sect. 241(d); 5 U.S.C. Sect. 552a; 44 U.S.C. Sect. 3501 et seq.

§
https://wwwn.cdc.gov/nchs/data/nhanes/2017-2018/manuals/2017_MEC_Interviewers_Procedures.pdf

¶ Research Use Only Linear Array HPV Genotyping Test which tests for HPV 6, 11, 16, 18, 26, 31, 33, 35, 39, 40, 42, 45, 51, 52[XR], 53, 54, 55, 56, 58, 59, 61, 62, 64, 66, 67, 68, 69, 70, 71, 72, 73, 81, 82, 83, 84, 89, and IS39.

** NHANES oversamples subpopulations to increase reliability and precision of estimates for specific racial/ethnic groups. Because of changes in oversampling methodology of persons who are Hispanic across the study period, the National Center for Health Statistics recommends that researchers do not report estimates for other Hispanic persons (i.e., Hispanic persons who are not Mexican American persons) using data before 2007 or for Hispanic subgroups other than Mexican American, in any survey cycle through 2018. Therefore, only estimates for persons who are Mexican American could be presented for this entire study period.

†† Anal, oral, or vaginal sex.

§§ Includes same sex and opposite sex partners.

¶¶ Prevalence estimates with a relative standard error of >30% were noted and considered unstable.

*** HPV 6, 11, 16, and 18.

††† HPV 31, 33, 45, 52, and 58.

§§§ Thirty-three HPV types detected using linear array that are not HPV 6, 11, 16, or 18.

¶¶¶ Twenty-eight types detected using linear array that are not HPV 6, 11, 16, 18, 31, 33, 45, 52, or 58.

****
https://wwwn.cdc.gov/nchs/data/nhanes/analyticguidelines/11-16-analytic-guidelines.pdf

††††
https://www.cdc.gov/rdc/index.htm
