# *Erratum:* Vol. 70, No. 12

**DOI:** 10.15585/mmwr.mm7013a6

**Published:** 2021-04-02

In the report “Declines in Prevalence of Human Papillomavirus Vaccine-Type Infection Among Females after Introduction of Vaccine — United States, 2003–2018,” on page 417, Table 1 contained alignment errors. The corrected table is as follows: 

**TABLE 1:** Prevalence of human papillomavirus (HPV) infection among females aged 14–34 years, by age group and survey years — National Health and Nutrition Examination Survey, United States, 2003–2018*

Age group (yrs) and HPV types	Prevaccine era 2003–2006	2007–2010	2011–2014	2015–2018	Comparison of 2015–2018 with 2003–2006	
	% (95% CI)				PR (95% CI)	aPR (95% CI)^†^
**14–19**	n = 1,363	n = 740	n = 797	n = 666	—	—
4vHPV^§^	11.5 (9.1–14.4)	5.0 (3.8–6.6)	3.3 (1.9–5.8)	1.1 (0.5–2.4)^¶^	0.10 (0.05–0.21)	0.12 (0.06–0.26)
Additional five types in 9vHPV**	8.4 (6.6–10.6)	6.1 (4.4–8.5)	5.3 (3.4–8.4)	2.3 (1.3–4.1)	0.28 (0.15–0.51)	0.35 (0.18–0.65)
Non-4vHPV^††^	31.2 (27.9–34.8)	25.3 (21.4–29.5)	25.5 (21.3–30.2)	20.9 (16.9–25.6)	0.67 (0.53–0.84)	0.72 (0.57–0.92)
Non-9vHPV^§§^	29.0 (26.0–32.2)	24.4 (20.8–28.4)	24.7 (20.6–29.4)	20.6 (16.6–25.3)	0.71 (0.57–0.90)	0.77 (0.61–0.98)
**20–24**	n = 432	n = 445	n = 442	n = 368	—	—
4vHPV^§^	18.5 (14.9–22.8)	19.9 (15.4–25.3)	7.2 (4.7–11.1)	3.3 (1.7–6.3)^¶^	0.18 (0.09–0.35)	0.19 (0.09–0.40)
Additional five types in 9vHPV**	16.5 (11.3–23.4)	13.8 (10.2–18.2)	13.2 (8.8–19.4)	10.2 (7.2–14.4)	0.62 (0.38–1.02)	0.62 (0.38–1.01)
Non-4vHPV^††^	50.7 (43.4–58.0)	57.4 (51.3–63.3)	55.8 (49.9–61.6)	49.9 (42.3–57.5)	0.98 (0.80–1.21)	0.97 (0.80–1.18)
Non-9vHPV^§§^	47.6 (40.7–54.6)	54.9 (48.9–60.8)	53.4 (47.8–58.8)	47.1 (39.7–54.7)	0.99 (0.80–1.22)	0.97 (0.79–1.18)
**25–29**	n = 403	n = 414	n = 395	n = 430	—	—
4vHPV^§^	11.8 (8.8–15.6)	13.1 (10.0–17.2)	8.8 (6.3–12.1)	9.1 (5.8–14.0)	0.77 (0.46–1.29)	0.85 (0.50–1.46)
Additional five types in 9vHPV**	10.8 (7.3–15.7)	13.1 (9.7–17.3)	13.9 (10.5–18.1)	11.6 (8.1–16.3)	1.07 (0.64–1.79)	0.99 (0.58–1.67)
Non-4vHPV^††^	43.8 (38.9–48.9)	48.6 (43.7–53.6)	43.7 (37.7–49.9)	45.2 (39.2–51.4)	1.03 (0.87–1.23)	1.05 (0.86–1.27)
Non-9vHPV^§§^	39.8 (34.8–45.0)	44.7 (40.0–49.4)	42.0 (36.2–48.0)	42.1 (36.6–47.9)	1.06 (0.88–1.27)	1.07 (0.88–1.31)
**30–34**	n = 389	n = 433	n = 433	n = 413	—	—
4vHPV^§^	9.5 (6.7–13.2)	8.9 (6.5–11.9)	7.1 (5.1–9.9)	6.2 (4.0–9.5)	0.65 (0.38–1.11)	0.67 (0.37–1.21)
Additional five types in 9vHPV**	9.8 (7.1–13.5)	6.8 (4.7–9.9)	6.9 (4.6–10.0)	6.9 (4.4–10.8)	0.70 (0.41–1.21)	0.68 (0.37–1.27)
Non-4vHPV^††^	44.5 (39.1–50.1)	37.8 (31.6–44.5)	39.2 (33.6–45.0)	34.7 (29.1–40.8)	0.78 (0.63–0.96)	0.82 (0.67–1.00)
Non-9vHPV^§§^	40.4 (35.0–46.0)	36.1 (30.3–42.3)	38.2 (32.7–44.0)	31.9 (26.6–37.6)	0.79 (0.64–0.98)	0.83 (0.67–1.03)

**Abbreviations**: aPR = adjusted prevalence ratio; CI = confidence interval; 4vHPV = quadrivalent HPV vaccine; 9vHPV = 9-valent HPV vaccine; PR = prevalence ratio.

* All analyses were weighted using the National Health and Nutrition Examination Survey examination sample weights.

^†^ Adjustments for aPR: females aged 14–19 years, race/ethnicity and ever had sex; females aged 20–24, 25–29, and 30–34 years, race/ethnicity and number of lifetime sex partners (fewer than three or three or more).

^§^ HPV 6, 11, 16, or 18.

^¶^ Relative standard error >30% and ≤50%, considered unstable.

** HPV 31, 33, 45, 52, or 58.

^††^ Thirty-three HPV types detected using linear array that are not HPV 6, 11, 16, or 18.

^§§^ Twenty-eight HPV types detected using linear array that are not HPV 6, 11, 16, 18, 31, 33, 45, 52, or 58.

